# High-sugar foods and breast cancer risk from the Health Examinees study

**DOI:** 10.1007/s00394-026-04134-0

**Published:** 2026-10-31

**Authors:** Woo-Kyoung Shin, Hyobin Lee, Dan Huang, Katherine De la Torre, Sukhong Min, Ji-Yeob Choi, Aesun Shin, Wonshik Han, Jung Eun Lee, Daehee Kang

**Affiliations:** 1 Division of Food and Pharmaceutical Technology, Mokwon University, Daejeon, 35349 Korea; 2 Department of Preventive Medicine, Seoul National University College of Medicine, Seoul, 03080 Korea; 3 Integrated Major in Innovative Medical Science, Seoul National University Graduate School, Seoul, 03080 Korea; 4 Department of Biomedical Sciences, Seoul National University Graduate School, Seoul, 03080 Korea; 5 Cancer Research Institute, Seoul National University, Seoul, 03080 Korea; 6 Departments of Surgery, Seoul National University Hospital, Seoul, 03080 Korea; 7 Biomedical Research Institute, Seoul National University Hospital, Seoul, 03080 Korea; 8 Department of Food and Nutrition, Seoul National University, Seoul, 08826 Korea

**Keywords:** Sugar intake, High-sugar foods, Breast cancer, The Health Examinees (HEXA) study, Prospective cohort study

## Abstract

**Purpose:**

The association between excessive sugar intake and breast cancer risk has been considered, however, results are unclear. This study aimed to examine the association between high-sugar foods intake and breast cancer in a prospective cohort study.

**Methods:**

This study included 92,314 women aged 40–69 years from the Health Examinees-Gem (HEXA-G) study (2004–2013). High-sugar foods were defined as sugar-rich food groups identified from the food frequency questionnaire. Multivariate hazard ratios (HRs) and 95% confidence intervals (CIs) for breast cancer risk according to high-sugar foods consumption were estimated using Cox proportional hazards models. A total of 713 breast cancer cases occurred over an average follow-up of 9.20 years.

**Results:**

The top three foods contributing to total sugar intake were fruits (42.89%), Korean traditional tea (5.60%), and ice cream (2.51%). Overall, consuming more than two servings of total high-sugar foods per week was associated with a 28% higher risk compared to less than once per month (HR = 1.28; 95% CI 1.02–1.61, *P* for trend = 0.05), with particularly significant associations observed for sugary cereals and ice cream. Notably, fruit intake, which accounted for the largest proportion of dietary sugar intake in this population, was not associated with breast cancer risk.

**Conclusion:**

Higher consumption of high-sugar foods was associated with an increased risk of breast cancer, whereas fruit intake was not. These findings suggest that sugar sources, rather than total sugar intake alone, may be important when considering dietary strategies for breast cancer prevention.

**Supplementary Information:**

The online version contains supplementary material available at https://doi.org/10.1007/s00394-026-04134-0.

## Introduction

The World Health Organization (WHO) has recommended reducing the consumption of free sugars or added sugars to less than 10% of total daily energy intake, recognizing its significant role in various health issues [1]. An umbrella review published in 2023 emphasized that high dietary sugar consumption generally has more harmful than beneficial effects on health [2]. Globally, sugar intake remains high, for instance in United States, 12.7% of total energy is contributed by added sugars [3]. In Asia, where carbohydrate-based diets are more common, the excessive intake of sugars presents particular health concerns. It is noteworthy that the daily sugar consumption in Korea highly exceeds the daily recommendation value [4].

Breast cancer is the leading cancer among women worldwide [5]. As part of cancer prevention strategies, understanding the contributing factors to breast cancer development is crucial. Genetic factors, lifetime estrogen exposure, and lifestyle factors such as regular exercise and alcohol consumption [6–10] are well-established contributors. Among these, in an umbrella review of meta-analyses, dietary factors have been increasingly recognized as an important determinant [11]. The systematic review conducted by World Cancer Research Fund have suggested that higher intake of fruits, vegetables, and foods rich in carotenoids or calcium may be associated with lower breast cancer risk [12], whereas evidence regarding whole grains, dietary fats, processed meat, and other dietary components remains less consistent [13–18].

Findings on the relationship between sugar intake and breast cancer risk are relatively limited. Most cohort studies, predominantly in Western populations, have examined sugar-sweetened beverage (SSBs) consumption [19–22], glycemic load (GL), or glycemic index (GI) [23–28] with breast cancer risk. However, relatively few studies have examined food-based sources of sugars, particularly distinguishing sugars derived from naturally occurring sources such as fruits from those derived from processed high-sugar foods containing added sugars. In addition, findings regarding total sugar intake or high-sugar foods and breast cancer risk have been inconsistent [29, 30].

Therefore, this study aims to investigate the association between high-sugar foods intake and breast cancer risk among Korean women, using data from the Health Examinees-Gem (HEXA-G) study. To our knowledge, this is the first study to examine this association in an Asian population.

## Methods

### Study population

The Health Examinees (HEXA) study is a large-scale, community-based prospective cohort study conducted in Korea from 2004 to 2013. The original cohort included 169,718 men and women aged 40–69 years who were recruited from 38 general hospitals and health examination centers in eight regions of Korea using a standardized protocol. Baseline data were collected through interviewer-administered questionnaires, physical examinations, laboratory tests, and biospecimen collection, as described previously [31, 32]. Trained interviewers administered a standardized questionnaire to collect data on socio-demographic characteristics, lifestyle behaviors, medical history, and dietary intake of participants, and reproductive history among women.

**Fig. 1:**
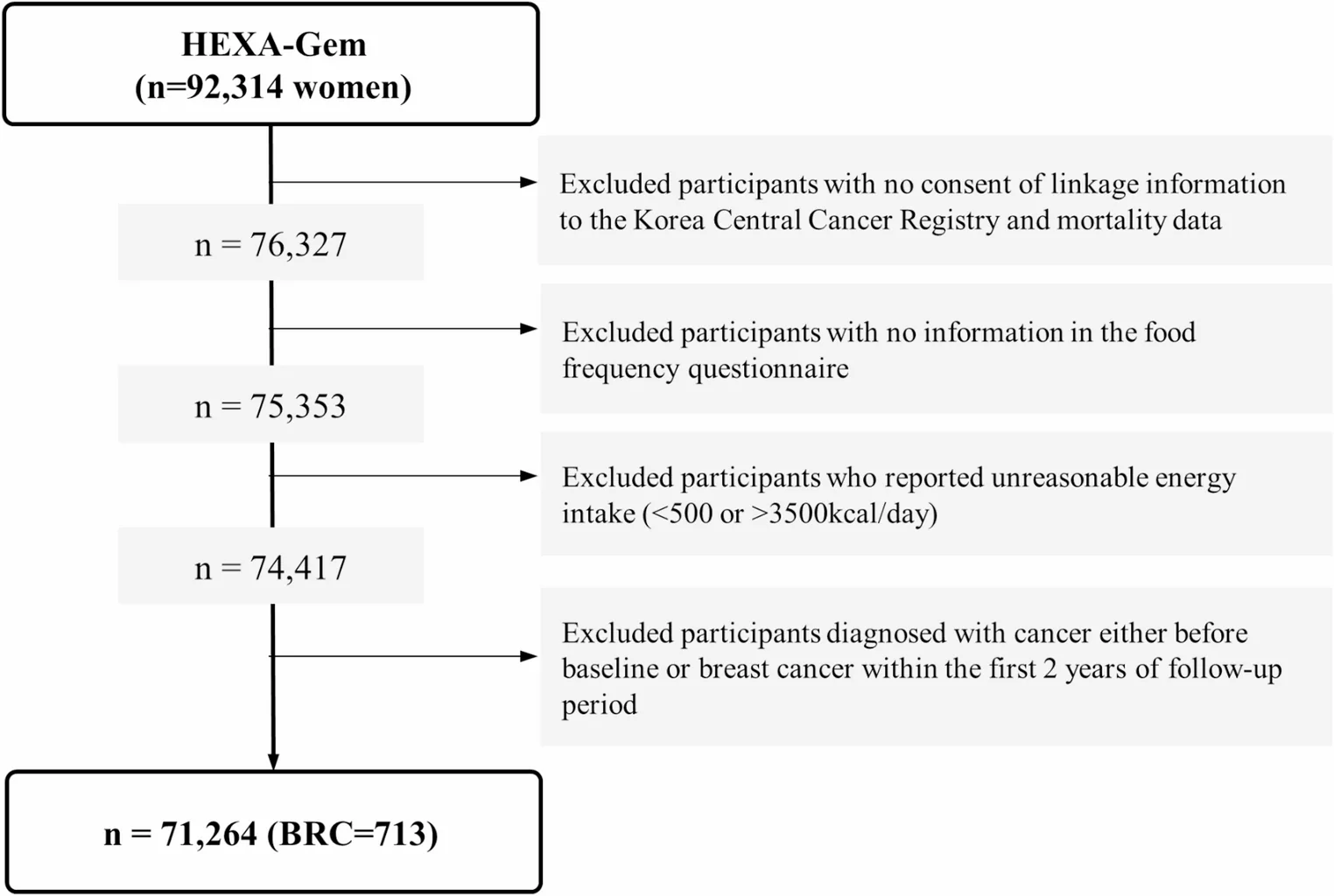
Flow chart of the study participants (*n* = 71,264), Health Examinees study, 2004–2013

For the present analysis, we used the HEXA-Gem, or HEXA-G dataset, an updated subset of the original HEXA cohort [31, 32]. The HEXA-G dataset excluded 21 of the 38 original study sites based on site-level eligibility criteria: sites that participated only in the pilot phase, sites that did not meet biospecimen quality control standards, and sites that discontinued participation within two years [33]. Consequently, 139,394 participants were included in the HEXA-G dataset before applying the exclusion criteria for the present study. This cohort study comprises 92,314 individuals enrolled at baseline. For the analysis, participants who did not consent to the linkage of cancer diagnosis data from the Korea Central Cancer Registry and death records from the Korean National Statistical Office (*n* = 15,987) and did not complete the food frequency questionnaires (FFQs) (*n* = 974) were excluded. In addition, participants with implausible total energy intake values (< 500 or > 3500 kcal/day for women; *n* = 936) were excluded. Also, participants diagnosed with any type of cancer prior to baseline enrollment (*n* = 3021) were subsequently excluded. Furthermore, women diagnosed with breast cancer within two years following baseline enrollment (*n* = 132) were excluded. After exclusion, a total of 71,264 women were included in the final analysis (Fig. 1). Women diagnosed with breast cancer within the first two years after baseline enrollment were excluded as a predefined eligibility criterion to reduce potential reverse causation due to preclinical disease that may have been present at baseline. Follow-up time for the remaining eligible participants was calculated from baseline.

The HEXA study is a component of the Korean Genome and Epidemiology Study (KoGES), and its protocol was reviewed and approved by the Ethics Committee of KoGES under the Korean National Institute of Health. Ethical approval for all study procedures was obtained from the Institutional Review Board (IRB) of Seoul National University Hospital (IRB No. E-2009-117-1159) and the Korea National Institute of Health (IRB No. 2014-08-02-3 C-A). Written informed consent was obtained from all participants at enrollment, including consent for linkage to cancer registry data from the Korea Central Cancer Registry and mortality data from the Korean National Statistical Office.

### Assessment of dietary intake

Dietary intake was assessed with an interviewer-administered semi-quantitative food frequency questionnaire (FFQ) in the HEXA study, which included 106 food items. The FFQ used nine categories for consumption frequency and a three categories for consumption amounts. The reliability and validity of the FFQ have been assessed in a previous study [34, 35]. The FFQ assessed usual dietary intake during the previous one year. For seasonal food items, including fruits, intake frequency was reported based on average consumption across seasons. Participants were asked about the frequency of consumption, ranging from “almost never” to “more than three times per day,” and portion sizes, which were categorized as one-half standard serving, one standard serving, and one and a half servings. The reported frequency and portion size of each food item were used to calculate the average daily intake of each item. The total high-sugar foods consumption was obtained by summing the serving/day values of each high-sugar food group. Total energy and nutrient intakes were estimated using the Food Composition Table provided in the Recommended Dietary Allowances for Koreans [36].

### Sugar content database

To estimate the total sugar intake of the Korean population, a comprehensive database of total sugar content was developed for all 106 food items included in the FFQ. This database was constructed using multiple national and international food composition databases and nutrition labeling sources, and was cross-validated across these sources to improve the accuracy and completeness of sugar content estimation. Detailed information on each data source is provided in Supplementary Table 1.

Using the constructed sugar content database for the 106 food items included in the FFQ, the sugar content of each item was converted to the amount of sugar per 1 gram of food. This value was then multiplied by each participant’s reported intake of the corresponding food item. Based on these calculations, individual sugar intake from each high-sugar food group, as well as total sugar intake from all foods, was estimated. High-sugar foods were operationally defined as FFQ food items or food groups characterized by relatively high sugar content and/or commonly consumed sources of dietary sugars in the Korean population. Based on these predefined criteria, seven high-sugar food groups were identified: sugary cereals; jam and butter; cakes and cookies; ice cream; sweets; carbonated drinks; and Korean traditional tea (sugar-based tea). Detailed classifications of the high-sugar food groups and their corresponding food items are presented in Table 1.

**Table 1:** Types of high-sugar food group

	High-sugar food group	Food items	Relative contribution to total sugar intake (%)
1	Korean traditional tea (Sugar-based tea)	Yuja tea (Korean Citrus tea), Maesil tea (Korean plum tea), Aloe, Sujeonggwa (Korean cinnamon punch), Ginseng tea, Sikhye (fermented rice punch), Jujube tea, Ssanghwa Tea (Korean medicinal herbal tea)	44.98
2	Ice cream	Ice cream	20.20
3	Carbonated drinks	Carbonated drinks (cola, sprite, etc.)	13.70
4	Cakes and cookies	Cakes/chocopie, cookie/cracker/snack	8.20
5	Sweets	Candy/chocolate/caramel	6.09
6	Jam and butter	Jam/honey/butter/margarine	2.13
7	Sugary cereals	Sugary cereals	1.03
	Total high sugar foods	Sugary cereals, cakes and cookies, ice cream, sweets, jam and butter, carbonated drinks, and Korean traditional tea	100.0

### Identification of breast cancer and follow-up of the cohort

Data regarding cancer diagnoses in the eligible cohort was acquired through linkage to the Korea Central Cancer Registry, utilizing the resident registration number, a unique identifier provided to all citizens and foreign residents. Data on cancer incidence up to December 31, 2018, were obtained from the National Cancer Center Korea. The primary outcome of this study was the incidence of breast cancer. Cancer incidence was defined according to the 10th revision of the International Classification of Diseases (ICD-10), with all cancers classified under code ‘C’ and breast cancer under code ‘C50’. Information on deaths was obtained through linkage to the Korean National Statistical Office database.

### Assessment of covariates

Information on smoking status, alcohol consumption status and frequency and amount (in glasses), highest education level, regular physical activity, parity, age at first birth, age at menarche, oral contraceptive use, and the presence of family history of breast cancer was collected through a structured questionnaire. At baseline, participants underwent physical examinations conducted by trained medical staff according to the standardized protocol of the Korea Centers for Disease Control and Prevention (KoGES), during which height (cm) and weight (kg) were measured. Body mass index (BMI, kg/m^2^) was derived by dividing body weight in kilograms by the square of height in meters.

### Statistical analysis

In this study, person-time for breast cancer incidence was calculated from the baseline date to the earliest occurrence of breast cancer diagnosis, death, or the end of the follow-up period (December 31, 2018).

For the descriptive analysis of participant characteristics, categorical variables were presented as frequencies and percentages, while continuous variables were summarized using means and standard deviations (SD). Comparisons of participants’ characteristics were conducted using analysis of variance (ANOVA) for continuous variables and chi-square tests for categorical variables.

To assess the contribution of individual food items to total sugar intake, we calculated the percentage of total sugar intake attributable to each item by dividing the amount of sugar provided by each food item by the total sugar intake.

For the Cox proportional hazards analyses, high-sugar food groups were categorized according to the distribution of intake in the study population to ensure sufficient numbers of participants in each category. Sugary cereals and carbonated drinks were classified into two categories; cakes and cookies, sweets, and jam and butter into three categories; and ice cream, Korean traditional tea, and total high-sugar foods intake into four categories.

The association between high-sugar foods consumption and breast cancer risk was evaluated using Cox proportional hazards models to estimate HRs and 95% confidence intervals (CIs). Given that age is a determinant of cancer outcomes, age was used as the time scale in the Cox models. Age at baseline was defined as the entry time to account for left truncation of the data [37], while age at cancer diagnosis, censoring, or last follow-up was defined as the exit time.

Multivariate hazard ratios (HRs) were estimated with adjustments for the following covariates: body mass index (BMI, kg/m^2^; continuous), total energy intake (kcal/day; continuous), education level (middle school or less, high school or college, undergraduate or higher), regular physical activity (yes or no), alcohol consumption (never or ever), smoking status (never or ever), parity (nulliparous, 1, 2, more than three), age at first birth (nulliparous, aged less than 25 years at first birth, aged 25 or more at first birth), age at menarche (aged less than 15, aged 15, aged 16 or more), oral contraceptive use (never or ever) and family history of breast cancer (yes or no). Covariates included in the multivariable models were selected based on established breast cancer risk factors and previous epidemiologic literature.

To assess linear trends, the median value of each exposure category was assigned and modeled as a continuous variable, and the corresponding coefficient was evaluated using the Wald test.

We examined whether the association differed by baseline age group (< 50 vs. ≥ 50 years), BMI (< 25 vs. ≥ 25 kg/m^2^), alcohol consumption (never vs. ever drinkers), and physical activity (no regular exercise vs. regular exercise). Effect modification was assessed by including cross-product interaction terms in the model, and statistical significance was evaluated using the likelihood ratio test, comparing models with and without the interaction terms. Missing values for covariates were handled by assigning the median value (for continuous variables) or the most frequent category (for categorical variables). The overall proportion of missing values was low (≤ 1% for most covariates), except for family history of breast cancer (9.8%).

All statistical analyses were performed using SAS software version 9.4 (SAS Institute Inc., Cary, NC, USA), with a two-sided significance level at 0.05.

## Results

Over 655,578 person-years of follow-up (mean follow-up duration: 9.20 years) among 71,264 participants, a total of 713 new cases of breast cancer were identified and included in the analysis.

**Fig. 2:**
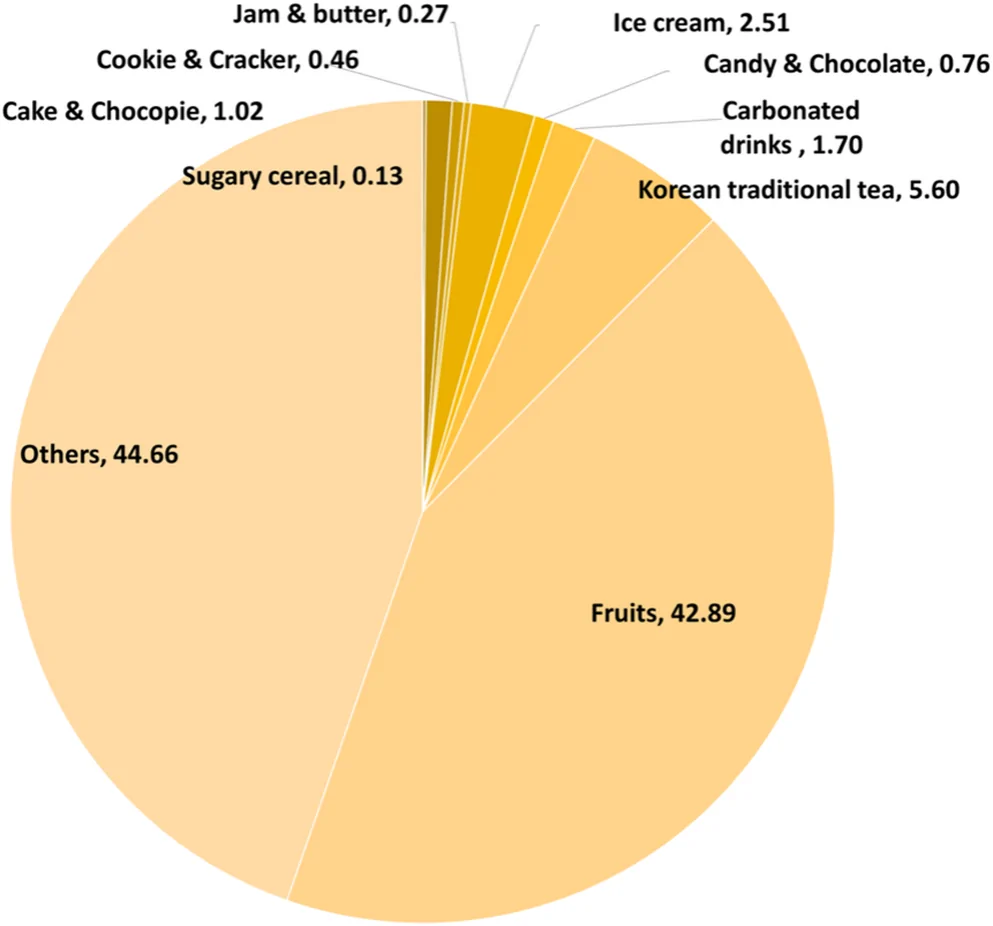
Relative contribution of each food group to total sugar intake (%)

Fruits were the largest contributors to total sugar intake (43%), whereas traditional Korean beverages, ice cream, and carbonated drinks contributed substantially to sugars from high-sugar foods (Fig. 2). Detailed contributions of fruit sources are presented in Supplementary Fig. 1.

The average age at baseline of participants was 52 years, and the mean total sugar intake was 44 g per day. Table 2 and Supplementary Table 3 shows the demographic characteristics and dietary intake according to high-sugar foods consumption. Women who consumed higher amounts of high-sugar foods were more likely to be younger, to drink alcohol, to be non-smokers, and to have an undergraduate degree or higher.

**Table 2:** Baseline characteristics of participants according to total high-sugar foods consumption, Health Examinees study, 2004–2013

Variables^1^		Total high-sugar foods^3^ consumption				
	Total		≤ 1/month	2–4/month	2/week	2/week <
No. of participants	71,264		17,626	18,007	17,817	17,814
Age (years)	52 (8)		54 (8)	53 (8)	51 (8)	51 (8)
BMI (kg/m^2^)	24 (3)		24 (3)	24 (3)	24 (3)	23 (3)
Education level						
Middle school or less	25,366 (36)		8202 (47)	6927 (38)	5444 (31)	4793 (27)
High school or college	30,789 (43)		6760 (38)	7738 (43)	8065 (45)	8226 (46)
Undergraduate school or more	14,409 (20)		2444 (14)	3162 (18)	4156 (23)	4647 (26)
Marital status						
Married or cohabiting	61,955 (87)		15,070 (86)	15,666 (87)	15,666 (88)	15,553 (87)
Single or divorce or widowed	9075 (13)		2489 (14)	2282 (13)	2100 (12)	2204 (12)
Regular exercise						
No	34,765 (49)		8852 (50)	8743 (49)	8691 (49)	8479 (48)
Yes	36,338 (51)		8730 (50)	9227 (51)	9088 (51)	9293 (52)
Alcohol consumption						
Never	47,468 (67)		12,426 (71)	12,181 (68)	11,462 (64)	11,399 (64)
Ever	23,519 (33)		5131 (29)	5761 (32)	6285 (35)	6342 (35)
Menopausal status						
Postmenopausal	41,985 (59)		12,200 (69)	11,116 (62)	9583 (54)	9086 (51)
Premenopausal	29,279 (41)		5426 (31)	6891 (38)	8234 (46)	8728 (49)
Parity						
Nulliparous	3447 (0)		609 (4)	866 (4)	624 (5)	1348 (6)
1	7293 (10)		1296 (8)	1921 (10)	1466 (11)	2610 (12)
2	39,984 (56)		7799 (50)	11,029 (56)	7917 (58)	13,239 (60)
≥ 3	20,205 (28)		5864 (37)	5960 (30)	3494 (26)	4887 (22)
Age at first birth						
Nulliparous	2556 (4)		442 (3)	626 (3)	452 (3)	1036 (5)
< 25 years	30,319 (43)		7952 (51)	8774 (44)	5392 (40)	8201 (37)
≥ 25 years	37,737 (53)		7112 (45)	10,296 (52)	7609 (56)	12,730 (57)
Age at menarche						
< 15 years	27,151 (38)		4723 (30)	7176 (36)	5527 (41)	9725 (44)
15 year	15,855 (22)		3202 (20)	4500 (23)	3133 (23)	5020 (23)
≥ 16 years	26,763 (38)		7350 (47)	7786 (39)	4664 (34)	6963 (31)
Oral contraceptive use						
Never	58,948 (83)		12,759 (82)	16,389 (83)	11,335 (84)	18,465 (83)
Ever	11,811 (17)		2770 (18)	3341 (17)	2146 (17)	3554 (16)
Family history of breast cancer						
No	63,254 (89)		15,678 (89)	16,008 (89)	15,819 (89)	15,749 (89)
Yes	1064 (2)		262 (2)	269 (2)	279 (2)	254 (1)
Total energy (kcal/day)	1681 (487)		1500 (4161)	1561 (423)	1681 (436)	1983 (520)
Protein intake (g/day) ^2^	57 (11)		57 (12)	57 (11)	57 (10)	57 (10)
Fat intake (g/day) ^2^	26 (10)		23 (10)	25 (10)	27 (10)	29 (10)
Carbohydrate intake (g/day) ^2^	301 (28)		306 (29)	304 (28)	299 (27)	297 (29)
Sodium intake (mg/day) ^2^	2405 (1189)		2513 (1348)	2395 (1219)	2378 (1120)	2334 (1040)
Fruit intake (g/day) ^2^	236 (205)		239 (224)	244 (215)	237 (198)	225 (179)
Vegetable intake (g/day) ^2^	121 (93)		124 (107)	121 (92)	118 (83)	120 (86)

^1^Continuous variables are reported as mean (standard deviation) value and categorical variables are reported as No. (%)

^2^Intakes of protein, fat, carbohydrate, sodium, fruit, vegetable, total sugar, liquid sugar, and solid sugar were energy adjusted using the residual method (Willet 2012)

We examined whether total and specific high-sugar foods were associated with breast cancer risk. Higher consumption of specific high-sugar foods was positively associated with breast cancer risk, whereas total sugar intake was not associated with breast cancer risk. Women who ate ice cream more than once per week had a 26% increased risk compared to non-consumers (HR = 1.26; 95% CI 1.00–1.58; *P* for trend = 0.03) (Table 3). Consumption of sugary cereal was associated with a higher risk (HR = 1.34; 95% CI 1.08–1.65; *P* for trend = 0.01; more than once every two months vs. never consumed). When considering total high-sugar foods intake—including sugary cereals, ice cream, and other high-sugar items—women who consumed more than two servings per week had a 28% greater risk of breast cancer compared to those consuming less than one serving per month (HR = 1.28; 95% CI 1.02–1.61; *P* for trend = 0.05). The association between breast cancer risk and sugar intake from total high-sugar foods showed a significantly elevated risk in the highest quartile, with an HR of 1.28 (95% CI 1.03–1.60) compared to the lowest quartile, and a significant trend across quartiles (*P* for trend = 0.04) (Table 4).

**Table 3:** Hazard ratios (HR) and 95% confidence intervals (CI) of breast cancer according to each high-sugar food consumption

		High-sugar food intake (serving)				
		1	2	3	4	*P* for trend ^2^
Sugary cereal	Serving	0	≥ 1/2 months			
	Numbers	63,370	7894			
	Cases	607	106			
	Person-years	584698.7	70879.74			
	HR (95% CI) ^1^	1.00 (ref.)	1.34 (1.08, 1.65)			0.01
Cakes and cookie	Serving	0	1/2months	≥ 1/month		
	Numbers	36,289	17,886	17,089		
	Cases	354	189	170		
	Person-years	333458.15	165276.7	156843.58		
	HR (95% CI) ^1^	1.00 (ref.)	0.99 (0.83, 1.19)	0.90 (0.74, 1.09)		0.26
Ice cream	Serving	0	≤ 1/month	1–3/month	≥ 1/week	
	Numbers	43,889	11,720	7689	7966	
	Cases	404	116	93	100	
	Person-years	400558.46	107875.21	72100.38	75044.39	
	HR (95% CI) ^1^	1.00 (ref.)	1.03 (0.84, 1.27)	1.21 (0.96, 1.52)	1.26 (1.00, 1.58)	0.03
Sweets	Serving	0	≤ 1/month	> 1/month		
	Numbers	41,336	13,437	16,491		
	Cases	405	140	168		
	Person-years	377482.08	123984.36	154111.99		
	HR (95% CI) ^1^	1.00 (ref.)	1.02 (0.84, 1.24)	0.98 (0.81, 1.17)		0.77
Jam and butter	Serving	0	1/2months	≥ 1/month		
	Numbers	48,368	2558	20,338		
	Cases	465	35	213		
	Person-years	445946.9	24186.47	185445.06		
	HR (95% CI) ^1^	1.00 (ref.)	1.30 (0.92, 1.84)	0.99 (0.83, 1.17)		0.81
Carbonated drinks	Serving	0	≥ 1/2 months			
	Numbers	55,301	15,963			
	Cases	552	161			
	Person-years	506597.08	148981.36			
	HR (95% CI) ^1^	1.00 (ref.)	0.97 (0.81, 1.16)			0.77
Korean traditional tea (Sugar based tea)	Serving	0	1/2months	1–2/month	≥ 3/month	
	Numbers	31,093	1370	21,466	17,335	
	Cases	295	10	223	185	
	Person-years	286760.11	13234.95	197179.28	158404.09	
	HR (95% CI) ^1^	1.00 (ref.)	0.71 (0.38, 1.33)	1.07 (0.90, 1.27)	1.07 (0.88, 1.30)	0.38
Total high-sugar foods ^3^	Serving	≤ 1/month	2–4/month	2/week	> 2/week	
(serving)	Numbers	17,626	18,007	17,817	17,814	
	Cases	147	171	189	206	
	Person-years	162350.39	165138.24	163180.05	164909.75	
	HR (95% CI) ^1^	1.00 (ref.)	1.11 (0.89, 1.38)	1.20 (0.96, 1.49)	1.28 (1.02, 1.61)	0.05

^1^HR (95% CI) : adjusted for BMI (kg/m^2^, continuous), energy intake (kcal/day, continuous), education level (middle school or less, high school or college, undergraduate or more), parity (nulliparous, 1, 2, more than three), age at first birth (nulliparous, aged < 25 years at first birth, aged ≥ 25 at first birth), age at menarche (< 15, 15, ≥ 16 years), oral contraceptive use (never, ever), regular exercise (no, yes), alcohol intake (never, ever), and the presence of a family history of breast cancer (no, yes)

^2^*P* for trend was obtained from a multivariate linear analysis where missing data values were substituted with median concentrations or common value by using the median value of each category as a continuous variable

^3^Total high-sugar food included sugary cereal, cake, cookie, jam, ice cream, sweets, soft drink, and sugar based tea

**Table 4:** Hazard ratios (HRs) and 95% confidence intervals (CIs) for breast cancer according to total sugar intake, and sugar intake from specific dietary sources

		Q1	Q2	Q3	Q4	*P* for trend ^2^
Total sugar intake ^3^	Numbers	17,824	17,818	17,811	17,813	
(g/day)	Cases	166	182	192	173	
	Person-years	163520.98	162982.62	162471.10	166603.74	
	HR (95% CI) ^1^	1.00 (ref.)	1.06 (0.85, 1.30)	1.09 (0.88, 1.34)	0.93 (0.75, 1.16)	0.44
Sugar intake from total high-sugar foods ^3, 4^	Numbers	17,813	17,819	17,822	17,810	
(g/day)	Cases	155	183	179	196	
	Person-years	164739.12	163330.05	163406.32	164085.42	
	HR (95% CI) ^1^	1.00 (ref.)	1.12 (0.90, 1.39)	1.12 (0.91, 1.40)	1.28 (1.03, 1.60)	0.04
Sugar intake from fruits ^4^	Numbers	17,820	17,822	17,809	17,815	
(g/day)	Cases	181	185	173	174	
	Person-years	164412.70	163621.75	162729.97	164814.01	
	HR (95% CI) ^1^	1.00 (ref.)	1.00 (0.82, 1.23)	0.92 (0.74, 1.13)	0.89 (0.72, 1.10)	0.19
Fruits ^4^	Numbers	17,821	17,816	17,819	17,808	
(serving/day)	Cases	184	177	170	182	
	Person-years	164509.99	162391.85	164659.55	163999.52	
	HR (95% CI) ^1^	1.00 (ref.)	0.94 (0.76, 1.15)	0.86 (0.70, 1.07)	0.89 (0.71, 1.10)	0.32
Sugar intake from solid foods ^4, 5^	Numbers	17,821	17,819	17,813	17,811	
(g/day)	Cases	178	179	184	172	
	Person-years	163093.77	162146.22	163419.24	166901.69	
	HR (95% CI) ^1^	1.00 (ref.)	0.97 (0.79, 1.19)	0.97 (0.79, 1.20)	0.88 (0.71, 1.08)	0.22
Sugar intake from liquid foods ^4, 6^	Numbers	17,823	17,823	17,808	17,810	
(g/day)	Cases	172	179	186	176	
	Person-years	164746.74	163417.32	162370.09	165026.77	
	HR (95% CI) ^1^	1.00 (ref.)	1.00 (0.81, 1.24)	1.03 (0.83, 1.27)	0.93 (0.75, 1.15)	0.49

^1^HR (95% CI) : adjusted for BMI (kg/m^2^, continuous), energy intake (kcal/day, continuous), education level (middle school or less, high school or college, undergraduate or more), parity (nulliparous, 1, 2, more than three), age at first birth (nulliparous, aged < 25 years at first birth, aged ≥ 25 at first birth), age at menarche (< 15, 15, ≥ 16 years), oral contraceptive use (never, ever), regular exercise (no, yes), alcohol intake (never, ever), and the presence of a family history of breast cancer (no, yes)

^2^*P* for trend was obtained from a multivariate linear analysis where missing data values were substituted with median concentrations or common value by using the median value of each category as a continuous variable

^3^High-sugar food include sugary cereal, cake, cookie, jam, ice cream, sweets, carbonated drinks, and sugar-based tea

^4^Intakes of food were energy adjusted using the residual method (Willet 2012)

^5^Sugars from solid food items, either naturally occurring or added during processing, Sugar from solid sources = Total sugar−Sugar from liquid sources

^6^Sugars from beverages and liquid sweeteners, (Milk, carbonated drinks, coffee, green tea, Korean traditional tea, pear/pear juice, apple/apple juice, orange/orange juice, grape/grape juice, tomato/cherry tomato/tomato juice)

No significant associations were observed for total sugar intake, sugar intake from fruits, or fruit intake with breast cancer risk. To examine whether high-sugar foods consumption was associated with other macronutrient intakes, we explored the associations between total carbohydrate and fat intake and breast cancer risk (Supplementary Table 4). No significant associations were observed across quartiles of carbohydrate intake (*P* for trend = 0.41) or fat intake (*P* for trend = 0.30).

The association between total high-sugar foods consumption and breast cancer risk was significant among women under the age of 50, with HRs of 1.50 (95% CI 1.05–2.15) in Q3 and 1.52 (95% CI 1.05–2.22) in Q4; however, the P for interaction was not significant (*p* = 0.48) (Table 5). This association was also observed among alcohol drinkers, where those in Q4 had a 49% higher risk compared to the reference group (HR = 1.49; 95% CI 1.01–2.21; *P* for trend = 0.04; *P* for interaction = 0.02). When stratified by BMI, the associations within each subgroup were not statistically significant; however, the interaction terms were significant (*P* for interaction = 0.04). Similar stratified analyses were conducted for sugary cereal and ice cream intake. Among women under the age of 50, higher consumption of sugary cereals was significantly associated with increased breast cancer risk (*P* for trend = 0.02; *P* for interaction = 0.44) (Supplementary Table 5). When stratified by BMI, the association between sugary cereal intake and breast cancer was significant in women with lower BMI (*P* for trend = 0.03; *P* for interaction = 0.01) (Supplementary Table 6). For ice cream, although the trend was not significant, significant interaction by BMI was observed (*P* for interaction = 0.02). Stratification by alcohol consumption revealed that the association between sugary cereal intake and breast cancer risk was significant in ever drinkers (*P* for trend = 0.02; *P* for interaction = 0.01), with a significant interaction also noted for ice cream (*P* for interaction = 0.04) (Supplementary Table 7). Additionally, when stratified by regular physical activity, the association between sugary cereal intake and breast cancer was significant among women who reported regular physical activity (*P* for trend = 0.01; *P* for interaction = 0.01), while ice cream intake showed a borderline significant trend in women with no regular physical activity (*P* for trend = 0.05) (Supplementary Table 8).

**Table 5:** Hazard ratios (HR) and 95% confidence intervals (CI) of breast cancer according to total high-sugar foods consumption by selected subgroups

		Total high-sugar foods^1^ intake (serving)					
		≤ 1/month	2–4/month	2/week	2/week <	*P* trend ^3^	*P* interaction ^4^
*Age at baseline*							
< 50 years	Numbers	4873	6186	7636	8298		
	Cases	43	67	101	107		
	HR (95% CI) ^2^	1.00 (ref.)	1.23 (0.84, 1.80)	1.50 (1.05, 2.15)	1.52 (1.05, 2.22)	0.06	
≥ 50 years	Numbers	12,753	11,821	10,181	9516		
	Cases	104	104	88	99		
	HR (95% CI) ^2^	1.00 (ref.)	1.06 (0.81, 1.40)	1.02 (0.76, 1.36)	1.15 (0.86, 1.55)	0.36	0.48
*BMI*							
< 25 kg/m^2^	Numbers	12,270	12,891	13,008	13,099		
	Cases	104	114	141	152		
	HR (95% CI) ^2^	1.00 (ref.)	1.01 (0.77, 1.31)	1.18 (0.91, 1.53)	1.22 (0.93, 1.59)	0.12	
≥ 25 kg/m^2^	Numbers	5349	5112	4805	4709		
	Cases	43	57	48	54		
	HR (95% CI) ^2^	1.00 (ref.)	1.38 (0.93, 2.06)	1.22 (0.80, 1.86)	1.43 (0.93, 2.21)	0.23	0.04
*Alcohol consumption*							
Never drink	Numbers	12,426	12,181	11,462	11,399		
	Cases	103	115	115	120		
	HR (95% CI) ^2^	1.00 (ref.)	1.11 (0.85, 1.48)	1.14 (0.87, 1.49)	1.14 (0.86, 1.51)	0.51	
Ever drink	Numbers	5131	5761	6285	6342		
	Cases	43	54	73	82		
	HR (95% CI) ^2^	1.00 (ref.)	1.10 (0.73, 1.64)	1.32 (0.90, 1.94)	1.49 (1.01, 2.21)	0.04	0.02
*Physical activity*							
No regular exercise	Numbers	8852	8743	8691	8479		
	Cases	73	87	84	99		
	HR (95% CI) ^2^	1.00 (ref.)	1.16 (0.85, 1.58)	1.07 (0.78, 1.48)	1.27 (0.91, 1.76)	0.20	
Regular exercise	Numbers	8730	9227	9088	9293		
	Cases	74	84	104	106		
	HR (95% CI) ^2^	1.00 (ref.)	1.06 (0.77, 1.45)	1.30 (0.96, 1.76)	1.26 (0.92, 1.74)	0.17	0.04

^1^Total high-sugar foods included sugary cereal, cake, cookie, jam, ice cream, sweets, soft drink, and sugar based tea

^2^HR (95% CI) : adjusted for BMI (kg/m^2^, continuous), energy intake (kcal/day, continuous), education level (middle school or less, high school or college, undergraduate or more), parity (nulliparous, 1, 2, more than three), age at first birth (nulliparous, aged < 25 years at first birth, aged ≥ 25 at first birth), age at menarche (< 15, 15, ≥ 16 years), oral contraceptive use (never, ever), regular exercise (no, yes), alcohol intake (never, ever), and the presence of a family history of breast cancer (no, yes)

^3^*P* for trend was obtained from a multivariate linear analysis where missing data values were substituted with median concentrations or common value by using the median value of each category as a continuous variable

^4^Interaction was calculated using Wald test of cross-product terms

## Discussion

In this large prospective cohort study of Korean women, we found that higher consumption of high-sugar foods was significantly associated with an increased risk of breast cancer. Specifically, the overall intake of high-sugar foods, as well as sugar intake from total high-sugar foods were both positively associated with breast cancer incidence. Also, frequent intake of sugary cereals and ice cream was associated with elevated risk. In contrast, total sugar intake and fruit intake were not associated with breast cancer risk.

In this study, women who consumed total high-sugar foods more than twice per week had a significantly higher risk than those with lower intake. Furthermore, sugar intake from total high-sugar foods was significantly associated with breast cancer risk, with higher risk observed in the highest versus lowest quartile. This finding aligns with a cohort study by Debras et al. [29], which observed a significant association between sugars from all sugary food groups excluding fruits and breast cancer risk. Also, higher intake of SSBs, a major source of added sugars, was associated with increased breast cancer risk in another cohort study [19].

Emerging evidence suggests that the association between added sugars consumption and breast cancer risk may involve multiple biological pathways. High intake of added sugars contribute to insulin resistance, leading to elevated circulating insulin and insulin-like growth factor-1(IGF-1) levels [38]. Supporting this, studies have reported positive associations between dietary GI or GL and breast cancer risk [24, 26–28]. Furthermore, mammographic breast density (MD), a known intermediate marker of breast cancer risk [39], has been positively associated with the intake of sweet foods and SSBs, as reported by Duchaine et al. [40].

Stratified analyses provided additional insight into the heterogeneity of associations between total high-sugar foods and breast cancer risk. Notably, a positive association between total high-sugar foods intake and breast cancer risk was observed only among women under 50 years. This age-specific finding should be interpreted cautiously, as age may reflect multiple biological and behavioral factors related to breast cancer risk. Although menopausal status may partly contribute to these differences, age should not be considered a direct surrogate for menopausal status, particularly because menopausal status may change during follow-up. Furthermore, the association was significant among current drinkers. Given that alcohol consumption is a known risk factor for breast cancer and alters estrogen metabolism, it is plausible that sugar and alcohol may exert synergistic effects through overlapping hormonal pathways such as insulin and IGF-1 pathways [41].

Although sugars from total high-sugar foods was significantly associated with breast cancer risk, total sugar intake, regardless of food source, was not. This finding aligns with Debras et al. [29], that reported sugars from sugary food groups excluding fruits was associated with increased breast cancer risk, whereas sugars from all sugary products was not. In our cohort, fruits accounted for 42.89% of total sugar intake, yet fruits intake, which is a source of natural sugars, was not associated with breast cancer risk. Similarly, a meta-analysis of Asian populations showed that fruit may reduce breast cancer risk [42]. These findings highlight the importance of the sugar source, suggesting that sugars from fruits may be associated with different metabolic effects than added sugars in processed foods [43, 44]. In addition, fruits contain various potentially protective components, including dietary fiber, carotenoids, polyphenols, vitamins, and other antioxidant compounds [45], which may counterbalance the potentially adverse metabolic effects of naturally occurring sugars.

A closer examination of high‑sugar food groups revealed that sugary cereals and ice cream were associated with increased breast cancer risk. For sugary cereals, despite the relatively low intake level in this cohort, the combination of refined grains and added sugars, often consumed at breakfast, may contribute to greater postprandial insulin responses [46]. However, the intake level of sugary cereals in this cohort was relatively low, and therefore the finding should be interpreted cautiously. In the case of ice cream, the dual impact of added sugars and saturated fat may influence hormonal metabolism, further contributing to breast cancer development [15, 47]. This aligns with Debras et al. [29], who reported that sugars from milk-based desserts was independently associated with increased risk.

This study has several strengths. To our knowledge, it is the first prospective cohort study in an Asian population to assess the association between high-sugar foods consumption and breast cancer risk. We utilized data from the HEXA study, the largest prospective cohort study in Korea, with validated cancer outcomes based on the Korean Central Cancer Registry. The long follow-up period of nearly 10 years, large sample size, and use of a validated FFQ increase the robustness of our findings. Also, in this study, we classified major sugar-contributing foods into natural sources (e.g., fruits) and high-sugar foods containing added sugars. We then examined the associations of individual high-sugar food items and total high-sugar foods intake with breast cancer risk. This approach enabled us to explore not only the quantity of sugar consumed but also the sugar sources may influence breast cancer risk. In addition to using a Korean-specific validated FFQ, we developed a comprehensive sugar content database tailored to Korean foods by referencing reliable sources, including national and international food composition databases.

Nevertheless, this study has some limitations. First, dietary intake was assessed once at baseline, which may have introduced measurement error. However, any misclassification is likely to be non-differential, as dietary data were collected before cancer diagnosis, minimizing recall bias. The FFQ was previously validated for the Korean population and included detailed items and visual aids for portion estimation, though inaccuracies in reporting of specific high-sugar food items cannot be entirely excluded. Second, our sugar database did not distinguish between naturally occurring and added sugars, and total sugar intake was assessed without specific information on added sugar content. To partly address this limitation, we analyzed total sugar content from processed high-sugar foods as a proxy for added sugars, although misclassification of added sugar exposure cannot be fully excluded. In addition, fruit intake, which represented the largest contributor to total sugar intake and a major source of naturally occurring sugars, was not associated with breast cancer risk, providing indirect support that the observed associations may be driven by processed sugar-rich foods rather than total sugar intake alone. Third, we lacked detailed clinical data on breast cancer characteristics, such as molecular subtypes or cancer stage at diagnosis, which limited our ability to perform stratified analyses by disease subtype or progression. Fourth, missing values were handled using simple imputation methods, and the proportion of missing data was generally low for most covariates, except for family history of breast cancer. Although this approach was unlikely to materially affect the overall findings, given the relatively low level of missingness for most variables, some residual bias related to the handling of missing data, particularly for family history of breast cancer, cannot be completely excluded. Fifth, although BMI was included as a covariate because it is an established breast cancer-related factor and is associated with dietary behaviors, BMI may also partially lie on the causal pathway between high-sugar food intake and breast cancer risk. Therefore, BMI may act as both a mediator and potential confounder in this relationship. Sixth, although exclusion of breast cancer cases diagnosed within the first two years after enrollment was intended to reduce potential reverse causation due to preclinical disease, this approach differs from a formal lag-time analysis in which follow-up begins after a predefined period for all participants. Consequently, some potential for bias related to this analytic approach cannot be completely excluded. Therefore, the findings should be interpreted with this limitation in mind. Finally, as with all observational studies, residual confounding may remain despite adjustment for established breast cancer risk factors, particularly from factors such as hormone replacement therapy and dietary factors including calcium-rich and carotenoid-containing foods.

In conclusion, our findings suggest that frequent consumption of total high-sugar foods, particularly those containing high sugars such as sugary cereals and ice cream, may be associated with increase the risk of breast cancer among Korean women. These findings highlight the need to evaluate not just the quantity, but also the source and context in which sugars are consumed. Public health strategies aimed at reducing the intake of processed high-sugar foods may help support breast cancer prevention efforts. Future research should incorporate repeated measures of dietary intake, detailed breast cancer subtype classification, and accurate menopausal status at diagnosis to further explore these associations.

## Supplementary Information

Below is the link to the electronic supplementary material.

Supplementary Material 1

## Data Availability

The data generated in this study are not publicly available because of inclusion of personal data that may potentially be sensitive to the participants, even though researchers are provided with an anonymized dataset that excludes resident registration numbers. Derived data supporting the findings of this study are available from the corresponding author upon request.

## References

[1] World Health Organization Guideline (2015) Sugars intake for adults and children. World Health Organization, Geneva, Switzerland, p 59

[2] Huang Y, Chen Z, Chen B, Li J, Yuan X, Li J et al (2023) Dietary sugar consumption and health: umbrella review. BMJ 381:e071609. 10.1136/bmj-2022-071609 PMC10074550 37019448

[3] DiFrancesco L, Fulgoni VL 3rd, Gaine PC, Scott MO, Ricciuto L (2022) Trends in added sugars intake and sources among U.S. adults using the National Health and Nutrition Examination Survey (NHANES) 2001–2018. Front Nutr 9:897952. 10.3389/fnut.2022.897952 PMC9434277 36061886

[4] Lee HSKS, Yon M, Kim D, Lee JY, Nam J, Park SJ, Yeon JY, Lee SK, Lee HY, Kwon OS, Kim CI (2014) Dietary total sugar intake of Koreans: based on the Korea National Health and Nutrition Examination Survey (KNHANES), 2008–2011. J Nutr Health 47:268–276

[5] Bray F, Laversanne M, Sung H, Ferlay J, Siegel RL, Soerjomataram I et al (2024) Global cancer statistics 2022: GLOBOCAN estimates of incidence and mortality worldwide for 36 cancers in 185 countries. CA Cancer J Clin 74(3):229–263. 10.3322/caac.21834 38572751

[6] De Silva S, Tennekoon KH, Karunanayake EH (2019) Overview of the genetic basis toward early detection of breast cancer. Breast Cancer (Dove Med Press) 11:71–80. 10.2147/BCTT.S185870 PMC6345186 30718964

[7] Travis RC, Key TJ (2003) Oestrogen exposure and breast cancer risk. Breast Cancer Res 5(5):239–247. 10.1186/bcr628 PMC314432 12927032

[8] Bagnardi V, Rota M, Botteri E, Tramacere I, Islami F, Fedirko V et al (2015) Alcohol consumption and site-specific cancer risk: a comprehensive dose-response meta-analysis. Br J Cancer 112(3):580–593. 10.1038/bjc.2014.579 PMC4453639 25422909

[9] Singletary KW, Gapstur SM (2001) Alcohol and breast cancer: review of epidemiologic and experimental evidence and potential mechanisms. JAMA 286(17):2143–2151. 10.1001/jama.286.17.2143 11694156

[10] Chen X, Wang Q, Zhang Y, Xie Q, Tan X (2019) Physical activity and risk of breast cancer: a meta-analysis of 38 cohort studies in 45 study reports. Value Health 22(1):104–128. 10.1016/j.jval.2018.06.020 30661625

[11] Papadimitriou N, Markozannes G, Kanellopoulou A, Critselis E, Alhardan S, Karafousia V et al (2021) An umbrella review of the evidence associating diet and cancer risk at 11 anatomical sites. Nat Commun 12(1):4579. 10.1038/s41467-021-24861-8 PMC8319326 34321471

[12] World Cancer Research Fund Diet, nutrition, physical activity and breast cancer Continuous Update Project 2018

[13] Shin WK, Lee HW, Shin A, Lee JK, Lee SA, Lee JE et al (2020) Multi-grain rice diet decreases risk of breast cancer in Korean women: results from the health examinees study. Nutrients. 10.3390/nu12082273 PMC7468941 32751310

[14] Farvid MS, Chen WY, Rosner BA, Tamimi RM, Willett WC, Eliassen AH (2019) Fruit and vegetable consumption and breast cancer incidence: repeated measures over 30 years of follow-up. Int J Cancer 144(7):1496–1510. 10.1002/ijc.31653 PMC6440478 29978479

[15] Sieri S, Krogh V, Ferrari P, Berrino F, Pala V, Thiebaut AC et al (2008) Dietary fat and breast cancer risk in the European prospective investigation into cancer and nutrition. Am J Clin Nutr 88(5):1304–1312. 10.3945/ajcn.2008.26090 18996867

[16] Schulz M, Hoffmann K, Weikert C, Nothlings U, Schulze MB, Boeing H (2008) Identification of a dietary pattern characterized by high-fat food choices associated with increased risk of breast cancer: the European prospective investigation into cancer and nutrition (EPIC)-potsdam study. Br J Nutr 100(5):942–946. 10.1017/S0007114508966149 18377685

[17] Castello A, Rodriguez-Barranco M, Lope V, Guevara M, Colorado-Yohar S, Dorronsoro A et al (2024) High adherence to Western dietary pattern increases breast cancer risk (an EPIC-Spain study). Maturitas 179:107868. 10.1016/j.maturitas.2023.107868 37925868

[18] Pala V, Krogh V, Berrino F, Sieri S, Grioni S, Tjønneland A et al (2009) Meat, eggs, dairy products, and risk of breast cancer in the European prospective investigation into cancer and nutrition (EPIC) cohort. Am J Clin Nutr 90(3):602–612. 10.3945/ajcn.2008.27173 19491385

[19] Romanos-Nanclares A, Collins LC, Hu FB, Willett WC, Rosner BA, Toledo E et al (2021) Sugar-sweetened beverages, artificially sweetened beverages, and breast cancer risk: results from 2 prospective US cohorts. J Nutr 151(9):2768–2779. 10.1093/jn/nxab172 PMC8417930 34114021

[20] Arthur RS, Kirsh VA, Mossavar-Rahmani Y, Xue X, Rohan TE (2021) Sugar-containing beverages and their association with risk of breast, endometrial, ovarian and colorectal cancers among Canadian women. Cancer Epidemiol 70:101855. 10.1016/j.canep.2020.101855 33220638

[21] Romanos-Nanclares A, Toledo E, Gardeazabal I, Jiménez-Moleón JJ, Martínez-González MA, Gea A (2019) Sugar-sweetened beverage consumption and incidence of breast cancer: the Seguimiento Universidad de Navarra (SUN) project. Eur J Nutr 58(7):2875–2886. 10.1007/s00394-018-1839-2 30284064

[22] Chazelas E, Srour B, Desmetz E, Kesse-Guyot E, Julia C, Deschamps V et al (2019) Sugary drink consumption and risk of cancer: results from NutriNet-Santé prospective cohort. BMJ 366:l2408. 10.1136/bmj.l2408 PMC6614796 31292122

[23] Castro-Quezada I, Sánchez-Villegas A, Martínez-González M, Salas-Salvadó J, Corella D, Estruch R et al (2016) Glycemic index, glycemic load and invasive breast cancer incidence in postmenopausal women: the PREDIMED study. Eur J Cancer Prev 25(6):524–532. 10.1097/cej.0000000000000209 26633163

[24] Sieri S, Pala V, Brighenti F, Agnoli C, Grioni S, Berrino F et al (2013) High glycemic diet and breast cancer occurrence in the Italian EPIC cohort. Nutr Metab Cardiovasc Dis 23(7):628–634. 10.1016/j.numecd.2012.01.001 22497978

[25] Romieu I, Ferrari P, Rinaldi S, Slimani N, Jenab M, Olsen A et al (2012) Dietary glycemic index and glycemic load and breast cancer risk in the European prospective investigation into cancer and nutrition (EPIC). Am J Clin Nutr 96(2):345–355. 10.3945/ajcn.111.026724 22760570

[26] Larsson SC, Bergkvist L, Wolk A (2009) Glycemic load, glycemic index and breast cancer risk in a prospective cohort of Swedish women. Int J Cancer 125(1):153–157. 10.1002/ijc.24310 19319984

[27] Lajous M, Boutron-Ruault MC, Fabre A, Clavel-Chapelon F, Romieu I (2008) Carbohydrate intake, glycemic index, glycemic load, and risk of postmenopausal breast cancer in a prospective study of French women. Am J Clin Nutr 87(5):1384–1391. 10.1093/ajcn/87.5.1384 18469262

[28] Higginbotham S, Zhang ZF, Lee IM, Cook NR, Buring JE, Liu S (2004) Dietary glycemic load and breast cancer risk in the women’s health study. Cancer Epidemiol Biomarkers Prev 13(1):65–70. 10.1158/1055-9965.epi-03-0066 PMC4166477 14744735

[29] Debras C, Chazelas E, Srour B, Kesse-Guyot E, Julia C, Zelek L et al (2020) Total and added sugar intakes, sugar types, and cancer risk: results from the prospective NutriNet-Santé cohort. Am J Clin Nutr 112(5):1267–1279. 10.1093/ajcn/nqaa246 32936868

[30] Makarem N, Bandera EV, Lin Y, Jacques PF, Hayes RB, Parekh N (2018) Consumption of sugars, sugary foods, and sugary beverages in relation to adiposity-related cancer risk in the Framingham offspring cohort (1991–2013). Cancer Prev Res (Phila) 11(6):347–358. 10.1158/1940-6207.Capr-17-0218 PMC7225083 29674390

[31] Kim Y, Han B-G, Group K (2017) Cohort profile: the Korean genome and epidemiology study (KoGES) consortium. Int J Epidemiol 46(2):e20–e 10.1093/ije/dyv316 PMC5837648 27085081

[32] Health Examinees Study Group (2015) The health examinees (HEXA) study: rationale, study design and baseline characteristics. Asian Pac J Cancer Prev 16(4):1591–1597. 10.7314/apjcp.2015.16.4.1591 25743837

[33] Shin S, Lee H-W, Kim CE, Lim J, Lee J-k, Lee S-A et al (2017) Egg consumption and risk of metabolic syndrome in Korean adults: results from the health examinees study. Nutrients 9(7):687 10.3390/nu9070687 PMC5537802 28671590

[34] Ahn Y, Kwon E, Shim J, Park M, Joo Y, Kimm K et al (2007) Validation and reproducibility of food frequency questionnaire for Korean genome epidemiologic study. Eur J Clin Nutr 61(12):1435–1441 10.1038/sj.ejcn.1602657 17299477

[35] Kim J, Kim Y, Ahn YO, Paik HY, Ahn Y, Tokudome Y et al (2003) Development of a food frequency questionnaire in Koreans. Asia Pac J Clin Nutr 12(3):243–250 14505984

[36] Food Composition Table in (2000) Recommended Dietary Allowances for Koreans 7th ed. Korean Nutrition Society, Seoul, Korea

[37] Kom EL, Graubard BI, Midthune D (1997) Time-to-event analysis of longitudinal follow-up of a survey: choice of the time-scale. Am J Epidemiol 145(1):72–80 10.1093/oxfordjournals.aje.a009034 8982025

[38] Christopoulos PF, Msaouel P, Koutsilieris M (2015) The role of the insulin-like growth factor-1 system in breast cancer. Mol Cancer 14:43. 10.1186/s12943-015-0291-7 PMC4335664 25743390

[39] Rice MS, Bertrand KA, VanderWeele TJ, Rosner BA, Liao X, Adami HO et al (2016) Mammographic density and breast cancer risk: a mediation analysis. Breast Cancer Res 18(1):94. 10.1186/s13058-016-0750-0 PMC5031307 27654859

[40] Duchaine CS, Dumas I, Diorio C (2014) Consumption of sweet foods and mammographic breast density: a cross-sectional study. BMC Public Health 14:554. 10.1186/1471-2458-14-554 PMC4071328 24969543

[41] Al-Sader H, Abdul-Jabar H, Allawi Z, Haba Y (2009) Alcohol and breast cancer: the mechanisms explained. J Clin Med Res 1(3):125–131. 10.4021/jocmr2009.07.1246 PMC3318874 22493645

[42] Shin S, Fu J, Shin WK, Huang D, Min S, Kang D (2023) Association of food groups and dietary pattern with breast cancer risk: a systematic review and meta-analysis. Clin Nutr 42(3):282–297. 10.1016/j.clnu.2023.01.003 36731160

[43] Ravn-Haren G, Dragsted LO, Buch-Andersen T, Jensen EN, Jensen RI, Nemeth-Balogh M et al (2013) Intake of whole apples or clear apple juice has contrasting effects on plasma lipids in healthy volunteers. Eur J Nutr 52(8):1875–1889. 10.1007/s00394-012-0489-z 23271615

[44] Gonzalez JT (2024) Are all sugars equal? Role of the food source in physiological responses to sugars with an emphasis on fruit and fruit juice. Eur J Nutr 63(5):1435–1451. 10.1007/s00394-024-03365-3 PMC11329689 38492022

[45] Slavin JL, Lloyd B (2012) Health benefits of fruits and vegetables. Adv Nutr 3(4):506–. 10.3945/an.112.002154. 16 PMC3649719 22797986

[46] Kang X, Wang C, Lifang L, Chen D, Yang Y, Liu G et al (2013) Effects of different proportion of carbohydrate in breakfast on postprandial glucose excursion in normal glucose tolerance and impaired glucose regulation subjects. Diabetes Technol Ther 15(7):569–574. 10.1089/dia.2012.0305 PMC3709592 23594032

[47] Murillo-Ortiz B, Martinez-Garza S, Cardenas Landeros V, Cano Velazquez G, Suarez Garcia D (2017) Effect of reduced dietary fat on estradiol, adiponectin, and IGF-1 levels in postmenopausal women with breast cancer. Breast Cancer (Dove Med Press) 9:359–364. 10.2147/BCTT.S132602 PMC5447696 28579830

