# Supplementary material for: High-sugar foods and breast cancer risk from the Health Examinees study
Source: Eur J Nutr. 2026 Oct 31;65(8):291. doi: 10.1007/s00394-026-04134-0 (PMC13723681; doi:10.1007/s00394-026-04134-0)
Supplement: Supplementary file 1 [file 394_2026_4134_MOESM1_ESM.docx]

**Supplementary Fig. 1 Relative contribution of each fruits to total sugar intake from fruits(%)**

**Supplementary Table 1** **Data sources used for construction of the sugar composition database**

| **No.** | **Data Source** | **Institution / Country** | **Reference** |
| --- | --- | --- | --- |
| 1 | Sugar Database Compilation for Commonly Consumed Foods | Korea Health Industry Development Institute, Korea | [1] |
| 2 | Korean Food Composition Table | Rural Development Administration, Korea | [2] |
| 3 | Food Composition Database | U.S. Department of Agriculture (USDA), USA | [3] |
| 4 | Nutrition labeling on processed foods | Processed food companies, Korea | - |
| 5 | Japanese Food Composition Database on Sugars | Japan | [4] |
| 6 | Food and Nutrient Database | Ministry of Food and Drug Safety, Korea | [5] |
| 7 | National Food and Nutrition Management System for Restaurant Foods | Ministry of Food and Drug Safety, Korea | [5] |

**Supplementary Table 2. Sugar content of food items included in the high-sugar food groups in the FFQ**

|  | **High-sugar food group** | **Sugar content (g/100g)** |
| --- | --- | --- |
| 1 | Sugary cereals | 14.53 |
| 2 | Cakes and cookies | 33.43 |
| 3 | Ice cream | 17.41 |
| 4 | Sweets | 48.74 |
| 5 | Jam and butter | 50.42 |
| 6 | Carbonated drinks | 10.74 |
| 7 | Korean traditional tea (Sugar-based tea) | 11.09 |
|  | Total High sugar foods | 186.37 |

**Supplementary Table 3. Baseline characteristics of participants regarding sugar intake according to total high-sugar foods consumption, Health Examinees study, 2004-2013.**

|  |  | **Total high-sugar foods^3^ consumption** | | | | |
| --- | --- | --- | --- | --- | --- | --- |
| **Variables ^1^** | **Total** | | ≤**1/month** | **2-4/month** | **2/week** | **2/week <** |
| No. of participants | 71264 | | 17626 | 18007 | 17817 | 17814 |
| Total sugar intake (g/day) ^2^ | 43.51 (28.05) | | 32.89 (22.96) | 36.67 (24.02) | 42.13 (23.59) | 62.32 (31.16) |
| Sugar intake from liquid food ^2, 4^ | 19.68 (17.84) | | 14.44 (14.57) | 16.25 (15.23) | 18.32 (14.85) | 29.67 (21.63) |
| Sugar intake from solid food ^2, 5^ | 23.84 (14.72) | | 18.45 (12.11) | 20.42 (12.54) | 23.81 (12.57) | 32.64 (16.94) |
| % energy from sugar intake | 10.2 (5.13) | | 8.73 (5.10) | 9.39 (5.06) | 10.12 (4.66) | 12.57 (4.86) |
|  |  | |  |  |  |  |
| Sugar intake from sugary foods (g/day) |  | |  |  |  |  |
| Sugars from sugary cereals | 0.06 (0.33) | | 0.00 (0.01) | 0.01 (0.07) | 0.04 (0.15) | 0.17 (0.64) |
| Sugars from cakes and cookies | 0.64 (1.83) | | 0.03 (0.14) | 0.22 (0.45) | 0.65 (0.9) | 1.67 (3.28) |
| Sugars from ice cream | 1.09 (3.07) | | 0.04 (0.16) | 0.33 (0.77) | 1.06 (1.44) | 2.94 (5.47) |
| Sugars from sweets | 0.33 (1.00) | | 0.01 (0.04) | 0.11 (0.21) | 0.27 (0.4) | 0.93 (1.81) |
| Sugars from jam and butter | 0.12 (0.40) | | 0.05 (0.26) | 0.08 (0.33) | 0.12 (0.36) | 0.21 (0.56) |
| Sugars from carbonated drinks | 0.74 (3.82) | | 0.01 (0.09) | 0.16 (0.55) | 0.55 (1.12) | 2.24 (7.32) |
| Sugars from Korean traditional tea (sugar-based tea) | 2.44 (5.69) | | 0.07 (0.18) | 0.81 (1.10) | 1.44 (1.40) | 7.42 (9.61) |
| Sugars from total high-sugar foods | 5.30 (8.61) | | 0.16 (0.26) | 1.64 (1.05) | 4.01 (1.79) | 15.37 (12.24) |
| Sugars from fruits | 18.66 (18.81) | | 16.08 (17.19) | 17.51 (18.16) | 18.55 (17.88) | 22.5 (21.15) |

1 Continuous variables are reported as mean (standard deviation) value and categorical variables are reported as No. (%)

2 Intakes of protein, fat, carbohydrate, sodium, fruit, vegetable, total sugar, liquid sugar, and solid sugar were energy adjusted using the residual method (Willet 2012)

3 Total high-sugar food included sugary cereal, cake, cookie, jam, ice cream, sweets, soft drink, and sugar based tea

4 Sugars from beverages and liquid sweeteners, (Milk, carbonated drinks, coffee, green tea, Korean traditional tea, pear/pear juice, apple/apple juice, orange/orange juice, grape/grape juice, tomato/cherry tomato/tomato juice)

5 Sugars from solid food items, either naturally occurring or added during processing, Sugar from solid sources = Total sugar−Sugar from liquid sources

**Supplementary Table 4 Hazard ratios (HR) and 95% confidence intervals (CI) of breast cancer according to intake of carbohydrate and fat**

|  | **Carbohydrate intake ^2^** | | | |  |
| --- | --- | --- | --- | --- | --- |
|  | **Q1** | **Q2** | **Q3** | **Q4** | *P* for trend ^3^ |
| Numbers | 15656 | 19858 | 13573 | 22177 |  |
| Cases | 123 | 197 | 141 | 252 |  |
| Person-years | 1627901 | 163441 | 163994 | 165336 |  |
| HR (95% CI) ^1^ | 1.00 (ref.) | 0.99 (0.81, 1.22) | 1.07 (0.87, 1.32) | 1.08 (0.87, 1.33) | 0.41 |
|  | **Fat intake ^2^** | | | |  |
|  | **Q1** | **Q2** | **Q3** | **Q4** | *P* for trend ^3^ |
| Numbers | 17818 | 17815 | 17821 | 17810 |  |
| Cases | 176 | 181 | 180 | 176 |  |
| Person-years | 165743 | 164505 | 163341 | 161972 |  |
| HR (95% CI) ^1^ | 1.00 (ref.) | 0.96 (0.78, 1.19) | 0.93 (0.75, 1.15) | 0.90 (0.72, 1.11) | 0.30 |

1 HR (95% CI) : adjusted for BMI (kg/m^2^, continuous), energy intake (kcal/day, continuous), education level (middle school or less, high school or college, undergraduate or more), parity (nulliparous, 1, 2, more than three), age at first birth (nulliparous, aged <25 years at first birth, aged ≥25 at first birth), age at menarche (<15, 15, ≥16 years), oral contraceptive use (never, ever), regular exercise (no, yes), alcohol intake (never, ever), and the presence of a family history of breast cancer (no, yes)

2 Intakes of fat and carbohydrate were energy adjusted using the residual method (Willet 2012)

3 P for trend was obtained from a multivariate linear analysis where missing data values were substituted with median concentrations or common value by using the median value of each category as a continuous variable.

**Supplementary Table 5 Hazard ratios (HR) and 95% confidence intervals (CI) of breast cancer according to each sugary food group intake by age**

|  |  | **High-sugar food intake (serving)** | | | |  |  |
| --- | --- | --- | --- | --- | --- | --- | --- |
|  |  | **1** | **2** | **3** | **4** | *P*  trend ^2^ | *P*  interaction ^3^ |
| **Sugary cereal** | Serving | 0 | ≥1/2 months |  |  |  |  |
| <50 years | Numbers | 22439 | 4554 |  |  |  |  |
|  | Cases | 249 | 69 |  |  |  |  |
|  | HR (95% CI) ^1^ | 1.00 (ref.) | 1.40 (1.06, 1.83) |  |  | 0.02 |  |
| ≥50 years | Numbers | 40931 | 3340 |  |  |  |  |
|  | Cases | 358 | 37 |  |  |  |  |
|  | HR (95% CI) ^1^ | 1.00 (ref.) | 1.23 (0.87, 1.74) |  |  | 0.24 | 0.44 |
|  |  |  |  |  |  |  |  |
| **Ice cream** | Serving | 0 | ≤ 1/month | 1-3/month | ≥1/week |  |  |
| <50 years | Numbers | 14237 | 4873 | 3867 | 4016 |  |  |
|  | Cases | 142 | 61 | 62 | 53 |  |  |
|  | HR (95% CI) ^1^ | 1.00 (ref.) | 1.24 (0.92, 1.67) | 1.57 (1.16, 2.12) | 1.35 (0.97, 1.86) | 0.04 |  |
| ≥50 years | Numbers | 29652 | 6847 | 3822 | 3950 |  |  |
|  | Cases | 262 | 55 | 31 | 47 |  |  |
|  | HR (95% CI) ^1^ | 1.00 (ref.) | 0.89 (0.66, 1.19) | 0.86 (0.59, 1.25) | 1.23 (0.89, 1.69) | 0.31 | 0.64 |

1 HR (95% CI) : adjusted for BMI (kg/m^2^, continuous), energy intake (kcal/day, continuous), education level (middle school or less, high school or college, undergraduate or more), parity (nulliparous, 1, 2, more than three), age at first birth (nulliparous, aged <25 years at first birth, aged ≥25 at first birth), age at menarche (<15, 15, ≥16 years), oral contraceptive use (never, ever), regular exercise (no, yes), alcohol intake (never, ever), and the presence of a family history of breast cancer (no, yes)

2 P for trend was obtained from a multivariate linear analysis where missing data values were substituted with median concentrations or common value by using the median value of each category as a continuous variable.

3 Interaction was calculated using Wald test of cross-product terms.

**Supplementary Table 6 Hazard ratios (HR) and 95% confidence intervals (CI) of breast cancer according to each sugary food group intake by body mass index**

|  |  | **High-sugar food intake (serving)** | | | |  |  |
| --- | --- | --- | --- | --- | --- | --- | --- |
|  |  | **1** | **2** | **3** | **4** | *P*  trend ^2^ | *P*  interaction ^3^ |
| **Sugary cereal** | Serving | 0 | ≥1/2 months |  |  |  |  |
| BMI <25kg/m^2^ | Numbers | 45182 | 6086 |  |  |  |  |
|  | Cases | 430 | 81 |  |  |  |  |
|  | HR (95% CI) ^1^ | 1.00 (ref.) | 1.31(1.03,1.67) |  |  | 0.03 |  |
| BMI ≥25kg/m^2^ | Numbers | 18171 | 1804 |  |  |  |  |
|  | Cases | 177 | 25 |  |  |  |  |
|  | HR (95% CI) ^1^ | 1.00 (ref.) | 1.42(0.92, 2.19) |  |  | 0.11 | 0.01 |
|  |  |  |  |  |  |  |  |
| **Ice cream** | Serving | 0 | ≤ 1/month | 1-3/month | ≥1/week |  |  |
| BMI <25kg/m^2^ | Numbers | 31314 | 8455 | 5663 | 5836 |  |  |
|  | Cases | 288 | 84 | 63 | 76 |  |  |
|  | HR (95% CI) ^1^ | 1.00 (ref.) | 1.04 (0.81, 1.37) | 1.10 (0.84, 1.45) | 1.28 (0.98, 1.66) | 0.06 |  |
| BMI ≥25kg/m^2^ | Numbers | 12564 | 3260 | 2025 | 2126 |  |  |
|  | Cases | 116 | 32 | 30 | 24 |  |  |
|  | HR (95% CI) ^1^ | 1.00 (ref.) | 1.03 (0.69, 1.52) | 1.53 (1.02, 2.31) | 1.19 (0.76, 1.89) | 0.25 | 0.02 |

1 HR (95% CI) : adjusted for BMI (kg/m^2^, continuous), energy intake (kcal/day, continuous), education level (middle school or less, high school or college, undergraduate or more), parity (nulliparous, 1, 2, more than three), age at first birth (nulliparous, aged <25 years at first birth, aged ≥25 at first birth), age at menarche (<15, 15, ≥16 years), oral contraceptive use (never, ever), regular exercise (no, yes), alcohol intake (never, ever), and the presence of a family history of breast cancer (no, yes)

2 P for trend was obtained from a multivariate linear analysis where missing data values were substituted with median concentrations or common value by using the median value of each category as a continuous variable.

3 Interaction was calculated using Wald test of cross-product terms.

**Supplementary Table 7 Hazard ratios (HR) and 95% confidence intervals (CI) of breast cancer according to each sugary food group intake by alcohol consumption**

|  |  | **High-sugar food intake (serving)** | | | |  |  |
| --- | --- | --- | --- | --- | --- | --- | --- |
|  |  | **1** | **2** | **3** | **4** | *P*  trend ^2^ | *P*  interaction ^3^ |
| **Sugary cereal** | Serving | 0 | ≥1/2 months |  |  |  |  |
| Never drink | Numbers | 42581 | 4887 |  |  |  |  |
|  | Cases | 394 | 59 |  |  |  |  |
|  | HR (95% CI) ^1^ | 1.00 (ref.) | 1.26 (0.95, 1.67) |  |  | 0.11 |  |
| Ever drink | Numbers | 20543 | 2976 |  |  |  |  |
|  | Cases | 206 | 46 |  |  |  |  |
|  | HR (95% CI) ^2^ | 1.00 (ref.) | 1.47 (1.06, 2.04) |  |  | 0.02 | 0.01 |
|  |  |  |  |  |  |  |  |
| **Ice cream** | Serving | 0 | ≤ 1/month | 1-3/month | ≥1/week |  |  |
| Never drink | Numbers | 30037 | 7606 | 4825 | 5000 |  |  |
|  | Cases | 273 | 64 | 53 | 63 |  |  |
|  | HR (95% CI) ^1^ | 1.00 (ref.) | 0.89 (0.68, 1.17) | 1.12 (0.83, 1.51) | 1.27 (0.95, 1.68) | 0.08 |  |
| Ever drink | Numbers | 13687 | 4062 | 2839 | 2931 |  |  |
|  | Cases | 129 | 49 | 38 | 36 |  |  |
|  | HR (95% CI) ^1^ | 1.00 (ref.) | 1.23 (0.89, 1.72) | 1.31 (0.91, 1.90) | 1.23 (0.84, 1.80) | 0.24 | 0.04 |

1 HR (95% CI) : adjusted for BMI (kg/m^2^, continuous), energy intake (kcal/day, continuous), education level (middle school or less, high school or college, undergraduate or more), parity (nulliparous, 1, 2, more than three), age at first birth (nulliparous, aged <25 years at first birth, aged ≥25 at first birth), age at menarche (<15, 15, ≥16 years), oral contraceptive use (never, ever), regular exercise (no, yes), alcohol intake (never, ever), and the presence of a family history of breast cancer (no, yes)

2 P for trend was obtained from a multivariate linear analysis where missing data values were substituted with median concentrations or common value by using the median value of each category as a continuous variable.

3 Interaction was calculated using Wald test of cross-product terms.

**Supplementary Table 8 Hazard ratios (HR) and 95% confidence intervals (CI) of breast cancer according to each sugary food group intake by physical activity**

|  |  | **High-sugar food intake (serving)** | | | |  |  |
| --- | --- | --- | --- | --- | --- | --- | --- |
|  |  | **1** | **2** | **3** | **4** | *P*  trend ^2^ | *P*  interaction ^3^ |
| **Sugary cereal** | Serving | 0 | ≥1/2 months |  |  |  |  |
| No Regular Exercise | Numbers | 31047 | 3718 |  |  |  |  |
|  | Cases | 297 | 46 |  |  |  |  |
|  | HR (95% CI) ^1^ | 1.00 (ref.) | 1.19 (0.87, 1.64) |  |  | 0.28 |  |
| Regular Exercise | Numbers | 32191 | 4147 |  |  |  |  |
|  | Cases | 309 | 59 |  |  |  |  |
|  | HR (95% CI) ^1^ | 1.00 (ref.) | 1.45 (1.09, 1.93) |  |  | 0.01 | 0.01 |
|  |  |  |  |  |  |  |  |
| **Ice cream** | Serving | 0 | ≤ 1/month | 1-3/month | ≥1/week |  |  |
| No Regular Exercise | Numbers | 21454 | 5660 | 3753 | 3898 |  |  |
|  | Cases | 194 | 53 | 44 | 52 |  |  |
|  | HR (95% CI) ^1^ | 1.00 (ref.) | 0.97 (0.72, 1.32) | 1.16 (0.83, 1.62) | 1.35 (0.98, 1.85) | 0.05 |  |
| Regular Exercise | Numbers | 22350 | 6017 | 3925 | 4046 |  |  |
|  | Cases | 210 | 62 | 48 | 48 |  |  |
|  | HR (95% CI) ^1^ | 1.00 (ref.) | 1.07 (0.80, 1.42) | 1.22 (0.89, 1.68) | 1.17 (0.84, 1.61) | 0.26 | 0.06 |

1 HR (95% CI) : adjusted for BMI (kg/m^2^, continuous), energy intake (kcal/day, continuous), education level (middle school or less, high school or college, undergraduate or more), parity (nulliparous, 1, 2, more than three), age at first birth (nulliparous, aged <25 years at first birth, aged ≥25 at first birth), age at menarche (<15, 15, ≥16 years), oral contraceptive use (never, ever), regular exercise (no, yes), alcohol intake (never, ever), and the presence of a family history of breast cancer (no, yes)

2 P for trend was obtained from a multivariate linear analysis where missing data values were substituted with median concentrations or common value by using the median value of each category as a continuous variable.

3 Interaction was calculated using Wald test of cross-product terms.

**References**

1.	Lee H. Sugar database compilation for commonly consumed foods. Osong: Korea Health Industry Development Institute; 2015.

2.	Food Composition Table in Recommended Dietary Allowances for Koreans 7th ed. [Internet]. Korean Nutrition Society, Seoul, Korea 2000 [cited 2025.08.31.].

3.	USDA Food Composition Database [Internet]. [cited 2025.08.31.]. Available from: <https://fdc.nal.usda.gov/>

4.	Japanese Food Composition Database on Sugars [Internet]. [cited 2025.08.31.]. Available from: <https://fooddb.mext.go.jp/>

5.	Food and Nutrient Database [Internet]. [cited 2025.08.31.]. Available from: <https://various.foodsafetykorea.go.kr/nutrient/>.
